# Differential Expression of Serum Proteins in Rats with Allergic Asthma: A Study Based on the Nanoliter Two-Dimensional Liquid Chromatography Technique

**DOI:** 10.1155/2017/8015703

**Published:** 2017-04-11

**Authors:** Xingke Yan, Ao Zhang, Lu Yu, Cheng Chen, Haifu Cui

**Affiliations:** ^1^College of Acupuncture, Moxibustion and Tuina, Gansu University of Chinese Medicine, Lanzhou 730000, China; ^2^Jilin Provincial Jilin Hospital of Integrated Traditional Chinese and Western Medicine, Jilin 132021, China; ^3^Daqing Chinese Medicine Hospital, Daqing 163311, China; ^4^Hunchun Chinese Medicine Hospital, Hunchun 133300, China

## Abstract

*Objective*. To investigate the pathogenesis of allergic asthma via the nanoliter two-dimensional liquid chromatography (nano-2D-LC) technique.* Method*. 24 Wistar rats were randomized into 3 groups: the blank-control group (A), the restrained group (B), and the asthma model group (C). The nanoliter two-dimensional liquid chromatography (nano-2D-LC) technique was used to study the differential protein expressions of the serum in asthmatic rats.* Results*. Compared with the restrained group, the data from the asthma model group displayed a distinctive peak, that is, peak number 13 (94.731 min). The data also displayed three missing peaks in the asthma group, that is, peak number 1 (77.489 min), peak number 2 (78.418 min), and peak number 5 (80.533 min), suggesting that the corresponding peptides might be related to the pathogenesis of asthma. Compared with the blank-control group, the restrained group showed 4 new peaks, that is, peak number 2 (78.418 min), peak number 4 (79.398 min), peak number 5 (80.533 min), and peak number 7 (81.824 min). The restrained group also displayed a missing peak, that is, peak number 3 (78.804 min), indicating that those 5 polypeptides might be related to the binding-induced stress stimuli.* Conclusion*. The study suggests that the pathogenesis of allergic asthma is closely related to abnormal levels of proteins; however, future animal experiments should identify the specific protein expressions caused by stress factors.

## 1. Introduction

In 1995, the* Global Initiative for Asthma (GINA)*, a joint publication of the WHO and NIH, defined bronchial asthma as chronic airway inflammation involving eosinophils (EOS), mast cells (MC), T-lymphocytes, and many other inflammatory cells [1]. The proteome is the entire set of proteins produced or modified by an organism or system. It adapts over time according to distinct conditions, or stresses, that a cell or organism undergoes. Proteomics is an interdisciplinary domain that has benefited greatly from genetic information produced by the Human Genome Project [2]. Differential protein identification based on the chromatographic technique using high automation and high throughput has been applied in various aspects, for example, in the molecular markers, diagnoses, and treatments of respiratory diseases. Furthermore, studies about the proteomics of bronchial asthma have made many important achievements [3–5]. Based on our previous work [6], in this experiment, in order to research the pathogenesis of allergic asthma, nano-2D-LC was used to observe the fluctuation of protein peaks of low abundance polypeptide hydrolysates in the serum of allergy asthmatic rats.

## 2. Materials and Methods

### 2.1. Chemicals and Apparatus

*Chemicals*. Chemicals used were ovum albumin (OVA, A.R., obtained from Sigma), aluminum hydroxide ([Al(OH)_3_], A.R., obtained from Changchun BCHT Biotechnology Co.), 0.9% sodium chloride solution (obtained from Changchun BCHT Biotechnology Co.), pentobarbital sodium (obtained from Sigma), bovine serum albumin (BSA, A.R., obtained from Sigma), trypsin (obtained from Sigma, St. Louis, MO), acetonitrile (ACN, G.R., obtained from Fisher Scientific, Fairlawn), methanol (MET, G.R., obtained from Fisher Scientific, Fairlawn), formic acid (FA, G.R., obtained from Fluka), sodium dihydrogen phosphate (NaH_2_PO_4_, A.R., obtained from Beijing Chemical Works), ammonium chloride (NH_4_CL, A.R., obtained from Beijing Chemical Works), and aurum serum protein mini kit (732-6701, Bio-Rad). All other chemicals used in this study were of analytical grade.

*Apparatus*. The apparatus used were Agilent 1100 nanoupgraded multidimensional liquid analysis system (Agilent), Agilent 1100 Workstation (Agilent), ultrasonic cleaner (Jining Ultrasound Electronic Instrument Factory), GBC-Cintra 20 UV-visible spectrophotometer (GBC, Australia), PHS-3TC Precision digital pH meter (Shanghai Reaches Instrument Limited Company), and super water purifier (Milli-Q Gradient).

### 2.2. Protocol

#### 2.2.1. Animals and Grouping

Male Wistar rats weighing 130 ± 30 g were used in this experiment and provided by the* Experimental Animal Center of Jilin University* (Certificate number: SCXK [JI] 2007-0003). 24 Wistar rats were divided into 3 groups via the random number table: the blank-control group (A), the restrained group (B), and the asthma model group (C).

#### 2.2.2. Duplicating the Allergy Asthmatic Rat Model

In accordance with the model reference utilized [7], on the first day of the experiment, the rats of the allergic-asthma model group were intraperitoneally injected with 1.5 mL of saline solution, containing 0.25 g of OVA and 0.25 g of Al(OH)_3_, which caused the allergic reaction. On day 15, after being anaesthetized with pentobarbital sodium, rats from this group were fastened to the operating table, and then endotracheal intubation was conducted and OVA (0.25 g/kg) was injected into the external jugular vein to stimulate the asthma attack.

Rats of the restrained group were only fastened to the operating table and were not injected with any chemicals or solution. Rats of the blank-control group were neither fastened to the operating table nor injected with any chemicals or solution; these rats were not interfered with in any way.

#### 2.2.3. Detection of Differential Protein Expressions in the Serum

*(1) Preparation of Serum Samples.* In order to ensure that a sufficient amount of clean blood could be extracted, blood samples were taken from the hepatic portal vein, following which each rat was killed by cervical dislocation. For each rat, the blood sample was 4 mL. The samples were left standing at room temperature for 30 min and then centrifuged for 10 min at 4480 rpm, and then the supernatant was removed and preserved at 80°C. The aurum serum protein mini kit was used to remove the high-abundance albumin and IgG proteins in order to better resolve the low-abundance proteins. After all the samples were pooled within each group, samples of 400 *μ*L for each group were taken and had the pH value adjusted to 7.5–8 with 100 mmol/L NH_4_HCO_3_ aqueous solution and then boiled for 5 min. A 15% ACN solution was added to the sample, which was then cooled to room temperature; trypsin was then added in order to adjust the mass ratio of trypsin found within the sample to 40 : 1. The sample solution was left overnight in a water bath at 37°C, and formic acid was then added to change the pH value to between 3 and 4 in order to stop the enzymatic reaction. Finally, the sample solution was filtered through a 0.22 *μ*m filter before loading to the high-performance liquid chromatography (HPLC) device.

*(2) Separation of Serum Low-Abundance Protein Hydrolysate via Nano-2D-LC.* A volume of 10 *μ*L was loaded into the device once the protein's total volume was determined by the Bradford method.

The first-dimensional chromatographic column of Strong Cation Exchange (SCX) was Agilent ZORBAX Bio-SCX Series II (50 × 0.8 mm, 3 *μ*m). Mobile phase A was 5 mmoL/L NaH_2_PO_4_-1% ACN (pH = 4.0). Solution mobile phase B was 1 moL/L NH_4_CL solution (pH = 4.0). Six-step elution: consecutively elute the sample with volume fractions 0% B, 10% B, 20% B, 50% B, 80% B, and 100% B for 10 min, respectively. Flow rate was 10 *µ*L/min. Enrichment column: the C18 column was Agilent ZORBAX 300SB (50 × 0.3 mm, 5 *μ*m). As with mobile phase A, the preconcentration and desalination processes used a solution of 5 mmoL/L NaH_2_PO_4_-1% ACN (pH = 4.0).

Nanoliter analytical RPLC's second-dimensional column: the C18 column was Agilent ZORBAX SB (150 × 0.5 mm, 5 *μ*m). Mobile phase A was 0.1% formic acid solution. Mobile phase B was acetonitrile-0.1% formic acid solution. The linear gradient elution process was as follows: V(A) : V(B) = 99 : 1, 99 : 1, 50 : 50, 1 : 99, 1 : 99, 99 : 1, 99 : 1 (0-5-20-25-35-45-50 min), with flow rate of 4 *μ*L/min, using a multiwavelength detector (MWD) with a detection wavelength of 254 nm.

#### 2.2.4. Data Analysis

SPSS 11.0 statistical software was used to process the data, which was expressed as mean ± SD. One-way ANOVA was used in the simultaneous comparison of the 3 groups. LSD method was used for instances of equal variances, and Dunnett T3 was used for any unequal variances. A result of *P* < 0.05 was considered to be statistically significant.

## 3. Results

**Figure 1:**
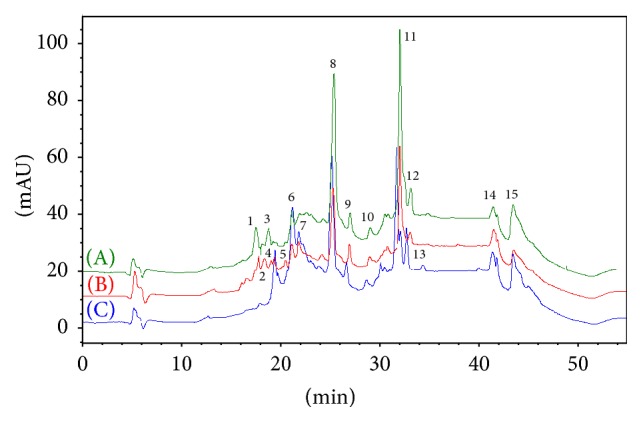
The peaks of peptides in the serum of 3 groups. (A) Blank-control group, (B) restrained group, and (C) asthma model group.

**Figure 2:**
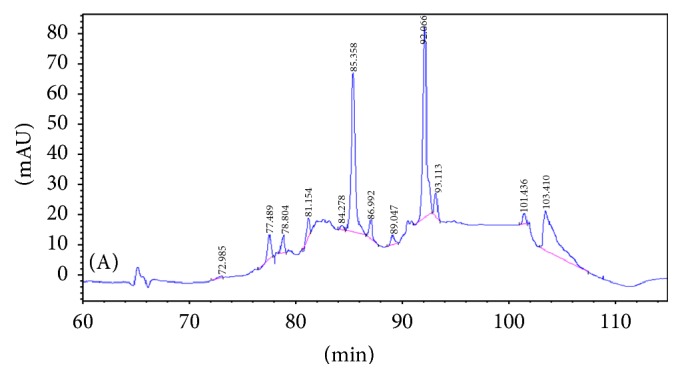
The peaks of peptides in the serum of blank-control group.

**Figure 3:**
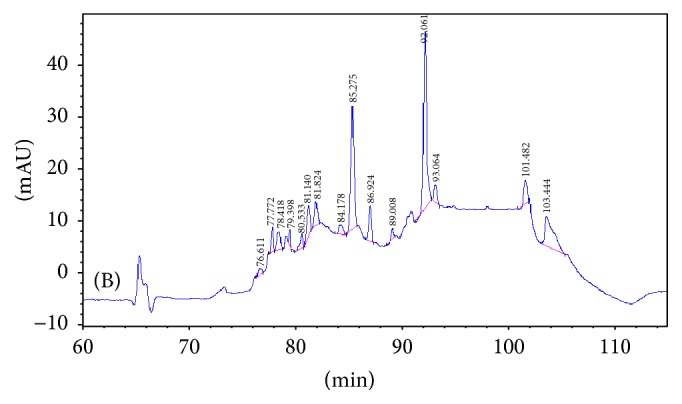
The peaks of peptides in the serum of restrained group.

**Figure 4:**
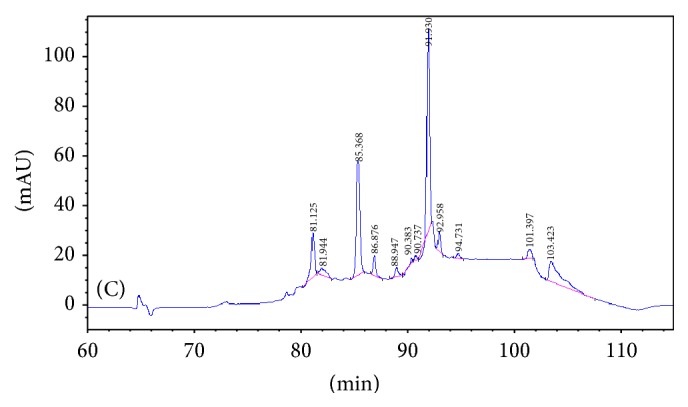
The peaks of peptides in the serum of asthma model group.

In order to identify the different peaks and those enzymatic hydrolysates displaying varying peak areas of common peak shapes, the peak area of the enzymatic hydrolysates from the asthma model group was compared with the two other groups (as shown in Figures 1–4 and Table 1). The results were as in Figures 1–4 and Table 1.

## 4. Discussions

### 4.1. Comparison of Protein Peaks among the Three Groups

(1) Three groups share 8 common peaks: peak number 6 (81.154 min), peak number 8 (85.358 min), peak number 9 (86.992 min), peak number 10 (89.047 min), peak number 11 (92.066 min), peak number 12 (93.113 min), peak number 14 (101.436 min), and peak number 15 (103.410 min).

(2) Comparing the restrained group with the blank-control group, 4 new peaks appeared: peak number 2 (78.418 min), peak number 4 (79.398 min), peak number 5 (80.533 min), and peak number 7 (81.824 min). This suggests that there might be a correlation between these 4 polypeptides and the binding-induced stress stimuli. Among the 9 common peaks, peak number 1 displayed a significant difference in the peak area (*P* < 0.01), suggesting a correlation between this polypeptide and the binding-induced stress stimuli.

(3) Comparing the asthma model group with the restrained group, 1 new specific peak appeared: peak number 13 (94.731 min). Three peaks lost their specificity: peak number 1 (77.489 min), peak number 2 (78.418 min), and peak number 5 (80.533 min), signifying that these polypeptides are related to the pathogenesis of allergic asthma.

There were a total of 10 common peaks, among which peak number 12 displayed significant variation on the peak area (*P* < 0.05) from the other 9 peaks, implying that this polypeptide is related to the pathogenesis of allergic asthma. The other 9 common peaks may have no relation to allergic asthma.

### 4.2. Analysis of Serum Protein Peak Expression in Asthmatic Rats

The deciphering of the human genetic code opened the next chapter in the scientific study of human beings; nonetheless, current knowledge of the human genome is still unable to explain the mechanism of the genome's compiled protein [8]. Therefore, proteomics has become one of the most important ways to study gene function and is now the main pillar of functional genomics [9]. At present, there is a lack of technology to synthesize proteins in vitro for proteomic research, for example, to identify polymerase chain reaction (PCR) used in genomics research [8]. Two-dimensional polyacrylamide gel electrophoresis (2-DE) utilizes the protein's isoelectric point and molecular weight to achieve the separation and selection of differentially expressed proteins, and it uses mass spectrometry to identify differential proteins. This technique has been widely applied in the research of respiratory diseases. However, there is a serious flaw in 2-DE: it cannot separate proteins when PI < 3 or PI > 12. Also, the operating procedure is complicated and the process is time-consuming and laborious. In recent years, high-performance liquid chromatographic separation technology has demonstrated a high rate of efficiency; though this technology can be used in the separation and purification of multicomponent substances, it is, however, more suitable in the analysis and identification of substances composed of a few materials: the discrimination effect is greatly reduced and automation and high throughput of proteomics research can be achieved [10, 11].

Nanoscaled two-dimensional capillary liquid chromatography is characterized by a straightforward operational procedure, efficient separation effect, high resolution, stability, and repeatability; consequently, it currently has wide application in the separation, purification, and preparation of polypeptides. As the flow rate is controlled at the nL/min level, the sample volume is significantly reduced, the mass spectrometry data acquisition time is extended, and the detection sensitivity is improved, which makes it more suitable for the separation and identification of peptide mixtures with only a few samples. Nano-2D-LC was thus used in this research to detect the low-abundance protein in the serum of rats with allergic asthma. Results indicated that the peptide peak at 79.398 min was the peak of asthma-related protein. Further mass spectrometric identification of retention-time effluent materials may lead to the identification of several enzymatic hydrolysate-related proteins associated with the onset of asthma in the serum.

Yuan and his team [12] applied surface-enhanced laser desorption and ionization time-of-flight mass spectrometry (SELDI-TOF-MS) to select the specific biomarkers in the serum of children with asthma; six highly expressed potential biomarkers were identified, with relative molecular weights of 5339, 5639, 5908, 4718, 5847, and 9300; nine low-expression potential biomarkers were identified, with relative molecular weights of 5164, 3767, 15314, 3656, 15135, 14978, 4178, 5219, and 3488. This method is capable of selecting several specific, potential biomarkers from the serum of asthmatic children. Aspirin-induced asthma (AIA) is a unique clinical syndrome, though its exact pathogenesis remains unclear. Using 2-DE and mass spectrometry, Lee et al.'s research [13] found six proteins to be differentially expressed in the plasma between patients with AIA and patients with ATA (aspirin-tolerant asthma) and also found eight proteins to have been significantly adjusted up or down once aspirin was ingested by patients with AIA. Plasma concentrations of C3a and C4a were higher in patients with AIA than in patients with ATA. After the ingestion of aspirin, though C3 decreased in both AIA patients and ATA patients, the concentration of C3a increased in the AIA patient group. Moreover, C3a and C4a levels and the ratios of C3a : C3 and C4a : C4 were directly correlated with changes to FEV1 values induced by the ingestion of aspirin.

At present, the study of asthma's mechanism is still a major obstacle in the medical field; compared to tumor research, it does not contain easily available target cells. The pathogenesis mechanism of asthma is complex. In etiology, it is closely related to genetics and the environment. In pathogenesis, it is related to the immune system, nervous system, and their mutual interactions. Finally, it is related to eosinophils, neutrophils, mast cells, and lymphocytes in pathophysiology. Through an analysis of the available literature, we found that asthma proteins are mostly associated with redox, airway's remodeling, anti-inflammation, and signaling pathways. Some types of proteins can be important biomarkers and therapeutic targets for asthma. Today, there are still many issues associated with the current application of proteomics research on bronchial asthma. However, through the combination of capillary electrophoresis, chromatography, and mass spectrometry and with the recent rapid development of protein Chip-TOF-MS technology, it is now possible to advance the study of asthma's biological mechanism from the perspective of proteomics and obtain new clinical diagnoses and treatment targets.

## Figures and Tables

**Table 1:** Areas of serum peptide peaks comparison in 3 groups (mean ± SD).

Number	Ret. time (min)	A (blank-control group)	B (restrained group)	C (asthma model group)
1	77.489	177.3077 ± 4.8170	64.64110 ± 7.1792^(2)^	
2	78.418		86.82484 ± 3.2402^(2)^	
3	78.804	65.57249 ± 48.1971		
4	79.398		37.60763 ± 8.8873^(2)^	230.9545 ± 124.0502
5	80.533		61.07169 ± 7.8572^(2)^	
6	81.154	225.9217 ± 173.4557	120.6287 ± 4.3967	254.2249 ± 136.8851
7	81.824		128.3332 ± 41.5655^(1)^	55.8214 ± 7.7610
8	85.358	1591.588 ± 1014.9047	614.3952 ± 162.0699^(1)^	1242.374 ± 320.6863
9	86.992	154.6185 ± 53.7288	124.6411 ± 15.6364	123.3418 ± 47.1595
10	89.047	76.36887 ± 16.2800	68.38598 ± 52.6746	89.51789 ± 29.1391
11	92.066	1056.652 ± 406.739	1033.819 ± 412.7315	1017.987 ± 794.5467
12	93.113	208.6571 ± 127.1982	49.48719 ± 6.9568^(1)^	174.6457 ± 67.7304^(3)^
13	94.731			48.7882 ± 7.4086^(4)^
14	101.436	95.77694 ± 15.2855	88.06084 ± 14.1367	107.5445 ± 20.0045
15	103.410	596.3253 ± 442.7393	267.3705 ± 61.2447	430.9182 ± 107.1599

^(1)^ *P* < 0.05, ^(2)^*P* < 0.01 versus blank-control group; ^(3)^*P* < 0.05, ^(4)^*P* < 0.01 versus restrained group.

## References

[1] Wasinger V. C., Cordwell S. J., Cerpa‐Poljak A. (1995). Progress with gene‐product mapping of the Mollicutes: Mycoplasma genitalium. *ELECTROPHORESIS*.

[2] Ferreira D., Seca A. M. L., Diana C. G. A., Silva A. M. S. (2016). Targeting human pathogenic bacteria by siderophores: a proteomics review. *Journal of Proteomics*.

[3] Mauri P., Riccio A. M., Rossi R. (2014). Proteomics of bronchial biopsies: galectin-3 as a predictive biomarker of airway remodelling modulation in omalizumab-treated severe asthma patients. *Immunology Letters*.

[4] O'Neil S., Sitkauskiene B., Babusyte A. (2012). Quantitative proteomics on bronchial biopsies from asthma and chronic obstructive pulmonary disease. *Allergy*.

[5] O'Neil S., Sitkauskiene B., Babusyte A. (2011). Coagulation pathway identified by proteomics in asthmatic bronchial biopsies: effects of treatment. *Allergy*.

[6] Yan X., Yu L., Chen C., Cui H. Differential protein expression profiling in serum of asthmatic rats on nano-2D-LC technique.

[7] Xingke Y., Guangquan Z., Yongqing Y. (2008). Effect of acupuncture on airway resistance and lung compliance in rats with allergic. *Jiangsu Journal of Traditional Chinese Medicine*.

[8] Venter J. C., Adams M. D., Myers E. W. (2015). The sequence of the human genome. *Science*.

[9] Wilson K. E., Ryan M. M., Prime J. E. (2004). Functional genomics and proteomics: application in neurosciences. *Journal of Neurology, Neurosurgery and Psychiatry*.

[10] Tentori A. M., Herr A. E. (2011). Photopatterned materials in bioanalytical microfluidic technology. *Journal of Micromechanics and Microengineering*.

[11] Okamoto A., Hasegawa T., Yamada K., Ohta M. (2011). Application of both high-performance liquid chromatography combined with tandem mass spectrometry shotgun and 2-D polyacrylamide gel electrophoresis for streptococcal exoproteins gave reliable proteomic data. *Microbiology and Immunology*.

[12] Yuan D., Shen C.-L., Jiang Z.-H. (2008). A screening study on serum biomarkers of children asthma by SELDI-TOF-MS. *Journal of Environmental & Occupational Medicine*.

[13] Lee S.-H., Rhim T. Y., Choi Y.-S. (2006). Complement C3a and C4a increased in plasma of patients with aspirin-induced asthma. *American Journal of Respiratory and Critical Care Medicine*.

